# Mutational Effects of Mobile Introns on the Mitochondrial Genomes of *Metschnikowia* Yeasts

**DOI:** 10.3389/fgene.2021.785218

**Published:** 2021-11-04

**Authors:** Scout R. L. Thompson, Dong Kyung Lee, Marc-André Lachance, David Roy Smith

**Affiliations:** Department of Biology, University of Western Ontario, London, ON, Canada

**Keywords:** endonuclease, homologous recombination, selfish elements, yeast, mobile intron

## Abstract

It has been argued that DNA repair by homologous recombination in the context of endonuclease-mediated cleavage can cause mutations. To better understand this phenomenon, we examined homologous recombination following endonuclease cleavage in a native genomic context: the movement of self-splicing introns in the mitochondrial genomes of *Metschnikowia* yeasts. Self-splicing mitochondrial introns are mobile elements, which can copy and paste themselves at specific insertion sites in mitochondrial DNA using a homing endonuclease in conjunction with homologous recombination. Here, we explore the mutational effects of self-splicing introns by comparing sequence variation within the intron-rich *cox1* and *cob* genes from 71 strains (belonging to 40 species) from the yeast genus *Metschnikowia*. We observed a higher density of single nucleotide polymorphisms around self-splicing-intron insertion sites. Given what is currently known about the movement of organelle introns, it is likely that their mutational effects result from the high binding affinity of endonucleases and their interference with repair machinery during homologous recombination (or, alternatively, via gene conversion occurring during the intron insertion process). These findings suggest that there are fitness costs to harbouring self-splicing, mobile introns and will help us better understand the risks associated with modern biotechnologies that use endonuclease-mediated homologous recombination, such as CRISPR-Cas9 gene editing.

## Introduction

The ability to safely modify the DNA of living organisms may soon be within our grasp thanks to CRISPR-Cas9. Despite its immense promise, CRISPR-Cas9 gene editing has not yet been approved for widespread therapeutic use because of its potential for introducing unwanted mutations (Kosicki et al., 2018). Recent reports have proposed that homologous recombination following endonuclease cleavage is responsible for CRISPR-Cas9’s on-target mutagenicity. This is because DNA-binding proteins, such as endonucleases, interfere with double-strand break repair by competing with the repair machinery for the target site (Reijns et al., 2015; Kaiser et al., 2016; Sabarinathan et al., 2016). Moreover, it has been shown that mutation rates are higher in regions of high recombination and that indels are often associated with a high density of single nucleotide polymorphisms (SNPs) (Lercher and Hurst, 2002; Tian et al., 2008).

An excellent model for studying the relationship between endonuclease activity and on-target mutagenicity in a native genomic context is the movement of self-splicing introns in mitochondrial genomes. Self-splicing introns are a class of mobile element commonly found in the mitochondrial DNAs (mtDNAs) of plants, fungi, and protists (Lambowitz and Belfort, 1993; Burger et al., 2003; Lang et al., 2007; Brown et al., 2014; Smith and Keeling, 2015; Megarioti and Kouvelis, 2020). These elements can copy and paste themselves at specific insertion sites in the host mitochondrial genome via their ability to form a complex secondary structure, which functions as a homing endonuclease (Cech et al., 1994; Lambowitz and Zimmerly, 2004). The homing endonuclease recognizes and binds to its target site with high affinity, inducing a double-stranded break (DSB). The break is then repaired by homologous recombination using the intron-containing strand as a template, resulting in intron movement (Cech et al., 1994; Lambowitz and Zimmerly, 2004).

Although self-splicing mitochondrial introns are found in a diversity of taxa (Del Vasto et al., 2015; Mower, 2020; Wideman et al., 2020), they are particularly prevalent within the yeast genus *Metschnikowia* (Lee et al., 2020), many members of which are commonly found in the guts of beetles inhabiting morning glories and other flowers around the world. *Metschnikowia* mtDNAs harbour among the largest numbers of organelle self-splicing introns of all eukaryotes. The *cox1* and *cob* genes from *Metschnikowia* mtDNAs are especially intron rich, with the number of introns varying from 0–20 within *cox1* and 0–13 within *cob*. These introns can be found at 25 and 13 unique intron insertion sites across *cox1* and *cob*, respectively (Supplementary Tables S1A, B), making this genus the largest known reservoir of self-splicing, mobile organelle introns (Férandon et al., 2010; Lee et al., 2020). For detailed summary statistics on the mitochondrial introns of *Metschnikowia* yeasts, including the variation in intron number across species/strains, the types of introns (e.g., group I or group II), and the presence/absence of intronic homing endonucleases, please refer to Supplemental Information of Lee et al. (2020).

**FIGURE 1:**
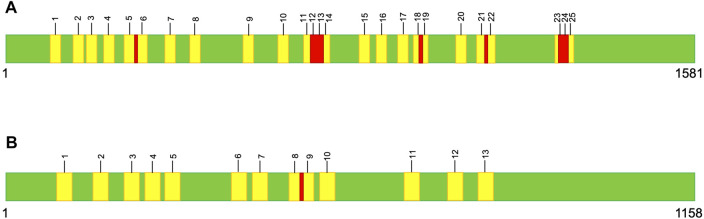
Graphical representation of the *cox1* **(A)** and *cob* **(B)** consensus sequences, which are 1,581 nt and 1,158 nt, respectively. Self-splicing-intron insertion sites are numbered and the 10 nt windows are highlighted in yellow. Regions where a single SNP occurred in two overlapping windows are highlighted in red.

Large variations in intron number can lead to remarkable differences in gene and genome size (Del Vasto et al., 2015; Mower, 2020; Wideman et al., 2020). Among *Metschnikowia* mtDNAs, there is a 30-fold size range for *cox1* and a 20-fold size range for *cob*, which encompasses the entire known size variation for both genes across the eukaryotic domain (Lee et al., 2020). More astonishing is that even within members of the same species, the size of *cox1* has been found to vary by up to 5 kb (Lee et al., 2020) — and keep in mind that the mature *cox1* mRNA from *Metschnikowia* species is less than 1.6 kb (Figure 1). *Metschnikowia* mitogenomes have other notable features, including extreme differences in intergenic content and genomic structure (e.g., linear vs circular mitochondrial chromosomes), both between and within species.

The co-occurrence of mtDNA diversity and large numbers of self-splicing introns in *Metschnikowia* may not be coincidental (Lee et al., 2020). If the movement of self-splicing introns is mutagenic, then this could help explain why *Metschnikowia* mitogenomes are so diverse. To explore this idea further, we studied the relationship between SNPs and their proximity to self-splicing-intron insertion sites within *cox1* and *cob* from 71 different species/strains of *Metschnikowia*. These two genes are ideal candidates for investigating this topic as they are both intron-dense and highly conserved, making sequence alignments and comparisons relatively straightforward (Chandrasekharan and Simmons, 2004; Linacre and Lee, 2005).

We predict that SNP abundance will increase close to self-splicing intron insertion sites in *cox1* and *cob*. If true, this would support the notion that the movement of mitochondrial introns is mutagenic and that this mutagenicity may be connected to endonuclease interference of homologous recombination or gene conversion. We should stress that this study is partly inspired by the work of Repar and Warnecke (2017) who found elevated levels of SNPs close to the insertion sites of self-splicing introns in the mitochondrial genomes of *Saccharomyces cerevisiae*, *Schizosaccharomyces pombe*, and *Lachancea kluyveri*. These findings ultimately led the authors to conclude that intron mobility is a direct driver of host genetic diversity.

## Materials and Methods

Annotated *Metschnikowia* mitochondrial genomes (Lee et al., 2020) were downloaded from GenBank (accessions MT421951-MT449704) and uploaded to Geneious Prime v. 2020.1.2 (Biomatter Ltd., Auckland, New Zealand). These genomes were generated as part of a large-scale collaborative effort to explore *Metschnikowia* diversity, natural history and phylogenetics (Lachance et al., 2016), and include *Metschnikowia* strains that have been collected intensively over 3 decades across a broad array of biogeographic zones (Lachance, 2011; de Oliveira Santos et al., 2015). The 71 *cox1* and *cob* genes were extracted, their introns were removed, and their coding sequences were aligned with MUSCLE (Edgar, 2004), implemented in Geneious using default settings. An initial pre-assessment of the alignments was performed to confirm that the coding regions and intron insertion sites were properly annotated. A few minor corrections were made, and the final alignments were consistent with previous analyses: 25 and 13 intron insertion sites across the *cox1* and *cob* alignments, respectively (Lee et al., 2020).

SNPs were evaluated in Geneious, but each polymorphic site within the alignments was also checked and counted manually. We used the chi-squared test of independence to determine if the number of SNPs within a given window was significantly greater than the number outside that window. To conduct this test, a series of 2x2 contingency tables for each window size were constructed in Excel for the *cox1* and *cob* alignments (Table 1). From the tables, the expected values were calculated, which were then used in the CHISQ.TEST function in Excel to obtain the *p*-values (Table 1).

**TABLE 1:** Statistical significance of the occurrence of SNPs near self-splicing intron insertion sites in the *cox1* and *cob* genes from 71 strains of *Metschnikowia*.

Window size	*cox1*			*cob*		
	*p*-value^a^	Observed^b^	Expected^b^	*p*-value^b^	Observed^b^	Expected^b^
1 nt	2.34E-55	610	340	0.12	151	134
5 nt	2.80E-14	1972	1,698	1.54E-3	744	670
10 nt (no overlap)	2.45E-10	3,687	3,397	2.84E-12	1,556	1,339
10 nt (overlap)	6.50E-21	3,897	3,463	1.18E-12	1,561	1,341

a Generated *p*-values of χ^b^ tests for significantly higher substitution rates near self-splicing intron insertion sites. *p*-values that were less than the alpha value (0.05) were deemed to be statistically significant.

b The differences between the observed and expected SNP count for the three window sizes around self-splicing intron insertion sites across the *cox1* and *cob* alignments.

## Results and Discussion

### Elevated SNP Density Around Mobile Intron Insertion Sites

To investigate the potential mutational effects of self-splicing intron movement in the mitogenomes of *Metschnikowia*, we recorded the number of SNPs in the *cox1* and *cob* genes across 71 species/strains. We binned the SNPs into the following window sizes: one nucleotide (nt), 5 nt, and 10 nt to the left and right of each intron insertion site. The number of SNPs that fell outside of these windows were also counted. Note: for the 10 nt windows, there were instances where a single SNP was present in two different windows (Figure 1). To account for this, two separate analyses were performed: one where every overlapping variant was counted twice and one where they were only counted once (Table 1).

For all parameters, except for the 1 nt window of the *cob* alignment, the observed *p*-values were less than 0.05 (Table 1), indicating that the null hypothesis (that there is no relationship between SNP density and self-splicing intron movement) could be rejected with confidence. To confirm that our findings were indicative of a higher density of SNPs within the chosen parameters (rather than lower), we calculated the observed versus the expected values (referring to the number of SNPs that we would expect to see within the windows under random conditions) (Table 1). We found that the observed values were higher than the expected values, confirming that there were more SNPs in these regions than predicted under a random model. Together, these data suggest that there is a relationship between mutagenicity and self-splicing intron movement, which may be connected to how homing endonucleases bind to their template strands while competing with mtDNA repair machinery (Reijns et al., 2015; Kaiser et al., 2016; Sabarinathan et al., 2016), or might be a consequence of gene conversion during the intron insertion process, which could affect the exonic nucleotides around insertion sites (Repar and Warnecke, 2017).

In simpler terms, we found that there was an average of 1.2x more SNPs in the *cox1* alignment and 1.5x more in the *cob* alignment within 10 nt of mobile intron insertion sites than throughout the rest of the sequences. While our values did achieve statistical significance (indicating that there are more SNPs in these areas than expected under random circumstances), they also indicate that there is significant variation outside of our selected windows as well, and that forces other than intron movement are likely contributing to the observed SNPs. Keeping in mind the huge reservoir of mitochondrial introns within this genus, there is a strong possibility that introns recently existed in other regions of the genes but have recently been lost. If true, this could have potentially led to some of the SNPs observed outside of the defined windows. We reiterate our call for more research on both the potential mutagenicity of self-splicing intron movement and the high amount of mtDNA variability exhibited by species of the genus *Metschnikowia*.

**FIGURE 2:**
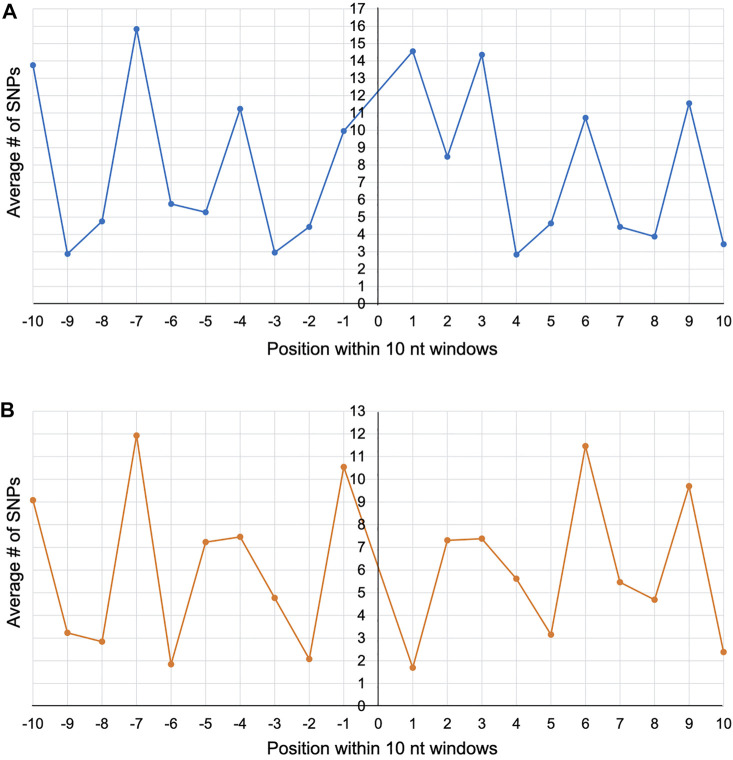
Line graphs depicting the average number of SNPs per nucleotide position within 10 nt windows around the self-splicing intron insertion sites across the *cox1* **(A)** and *cob* **(B)** alignments. The horizontal axis represents the nucleotide position (from -10 to +10) and the vertical axis represents the average number of SNPs.

To explore whether a particular site within the 10 nt window experienced a higher incidence of mutation, we plotted the average number of SNPs for each of the 20 nt positions (10 to the left and 10 to the right) across the 25 and 13 intron insertion sites of *cox1* and *cob*, respectively. Position 7 to the left of the insertion site showed the highest average number of SNPs for both gene alignments (Figures 2A,B), suggesting that this site may be particularly prone to mutations during intron movement. However, one should not place too much weight on this observation as there is obvious variability associated with the genetic code (i.e., the 3 nt periodicity), with introns inserting between codons (phase 0) occurring 68 and 69% of the time in *cox1* and *cob*, respectively.

### Consequences of Self-Splicing Introns

These findings raise an intriguing question: If the movement of self-splicing introns is mutagenic, why have *Metschnikowia* species accumulated so many mitochondrial introns? In other words, why would this genus retain something that arguably imposes a high mutational burden? The simplest answer is that *Metschnikowia* may not be able rid itself of these selfish elements. This could be a consequence of where the insertion sites are located as well as recurring horizontal gene transfer events involving the lateral exchange of mitochondrial introns between species and strains (Bhattacharya et al., 2001; Haugen et al., 2005).

Mitochondrial intron insertion sites tend to occur in highly conserved, constitutively expressed genes, like *cox1* and *cob* (Repar and Warnecke, 2017). Changes to the intron insertion sites of such genes can have deleterious consequences, meaning their sequences are typically maintained among close relatives. Self-splicing introns can, however, be lost from a genome through random deletion or gene conversion events (Goddard and Burt, 1999). But there is growing evidence of extensive horizontal gene transfer between yeast populations, meaning that even if an individual rids itself of a mitochondrial intron there is the potential that it will get it back from a neighbour (Bhattacharya et al., 2001; Haugen et al., 2005; Repar and Warnecke 2017).

There is also the possibility that *Metschnikowia* yeasts have become physiologically reliant on mitochondrial introns. Rudan et al. (2018) investigated two strains of *S. cerevisiae*: a wild-type strain with mobile introns and an artificial strain with the introns removed. They found that mitochondrial morphology was altered in the intron-less strain, resulting in a highly stressed cellular phenotype with lower fitness. It was proposed that these physiological changes were the consequence of overly efficient transcription of *cox1* and *cob* (their transcripts were in excess in the intron-less strain). One explanation for these observations is that mitochondrial introns can lead to inefficient splicing; thus, in genes where introns abound transcription needs to be upregulated, but removal of the introns can result in an overabundance of transcripts. This evolutionary lock-in event may partly explain the longstanding persistence of self-splicing introns in mitochondrial genomes, despite their potential deleterious effects.

Finally, it is noteworthy that the *Metschnikowia* species explored here are all insect symbionts (mostly of beetles). Although speculative, this symbiotic lifestyle could potentially reduce their reliance on proper mitochondrial function, as their insect vectors constantly make sure that they occupy the proper habitat. It may also greatly impact the population genetics of these yeasts, potentially increasing random genetic drift.

## Conclusion

We observed a higher density of SNPs near to self-splicing intron insertion sites, which we argue is the result of mitochondrial intron movement. The mutagenicity of self-splicing introns may result from incorrect mtDNA repair following homologous recombination due to the persistent presence and high binding affinity of endonucleases. These findings provide further support for the notion that, while once considered to have no ill effects (Werren, 2011), self-splicing introns impose a mutational burden on their host genomes (Mueller et al., 1996; Repar and Warnecke, 2017). We hope that this work will encourage more research on endonuclease binding and its potential interactions with host-cell repair machinery. A better understanding of this phenomenon could be crucial to treating diseases via CRISPR-Cas9 gene editing.

## Data Availability

The original contributions presented in the study are included in the article/Supplementary Material, further inquiries can be directed to the corresponding author.
