# Supplementary material for: Mutational Effects of Mobile Introns on the Mitochondrial Genomes of Metschnikowia Yeasts
Source: Front Genet. 2021 Nov 4;12:785218. doi: 10.3389/fgene.2021.785218 (PMC8601654; doi:10.3389/fgene.2021.785218)
Supplement: Supplementary file 1 [file DataSheet1.PDF]

Supplementary Table 1A: Location of mobile introns in the *cox1* gene of *Metschnikowia* yeast.

Mobile intron insertion site	Number of species with intron at site	Window range [1]		
		1 nt	5 nt	10 nt
1	36	111-112	107-116	102-121
2	20	166-167	162-171	157-176
3	26	195-196	191-200	186-205
4	57	234-235	230-239	225-244
5	31	285-286	281-290	276-295
6	5	311-312	307-316	302-321
7	60	375-376	371-380	366-385
8	13	437-438	443-442	428-447
9	32	561-562	557-566	552-571
10	8	640-641	636-645	631-650
11	22	693-694	689-698	684-703
12	49	706-707	702-711	697-716
13	63	717-718	713-722	708-727
14	47	732-733	728-737	723-242
15	21	824-825	820-829	815-834
16	60	864-865	860-869	855-874
17	47	915-916	911-920	906-925
18	22	949-950	945-954	940-959
19	51	960-961	956-965	951-970
20	38	1047-1048	1043-1052	1038-1057
21	54	1095-1096	1091-1100	1086-1105
22	38	1113-1114	11109-1118	1104-1123
23	8	1273-1274	1269-1278	1264-1283
24	8	1284-1285	1280-1289	1275-1294
25	29	1296-1297	1292-1301	1287-1306

[1] Range statistics based on the alignment of the coding sequences of 71 *Metschnikowia cox1* genes.

Supplementary Table 1B: Location of mobile introns in the *cob* gene of *Metschnikowia* yeast.

Mobile intron insertion site	Number of species with intron at site	Window range [1]		
		**1 nt**	**5 nt**	**10 nt**
1	41	99-100	95-104	90-109
2	36	159-160	155-164	150-169
3	46	212-213	208-217	203-222
4	16	247-248	243-252	238-257
5	6	277-278	273-282	268-287
6	55	393-394	389-398	384-403
7	52	429-430	425-434	420-439
8	60	489-490	485-494	480-499
9	36	507-508	503-512	498-517
10	21	541-542	537-546	532-551
11	51	684-685	680-689	675-694
12	38	756-757	752-761	747-766
13	53	807-808	803-812	798-817

[1] Range statistics based on the alignment of the coding sequences of 71 *Metschnikowia cob* genes.
